# Comparative Clinical Observation of Arthroscopic Microfracture in the Presence and Absence of a Stromal Vascular Fraction Injection for Osteoarthritis

**DOI:** 10.5966/sctm.2016-0023

**Published:** 2016-08-29

**Authors:** Phu Dinh Nguyen, Tung Dang‐Xuan Tran, Huynh Ton‐Ngoc Nguyen, Hieu Trung Vu, Phuong Thi‐Bich Le, Nhan Lu‐Chinh Phan, Ngoc Bich Vu, Ngoc Kim Phan, Phuc Van Pham

**Affiliations:** ^1^ 115 Hospital, Ho Chi Minh City, Vietnam; ^2^ Van Hanh General Hospital, Ho Chi Minh City, Vietnam; ^3^ Laboratory of Stem Cell Research and Application, University of Science, Vietnam National University, Ho Chi Minh City, Vietnam

**Keywords:** Osteoarthritis, Stromal vascular fraction, Platelet‐rich plasma, Arthroscopic microfracture

## Abstract

Osteoarthritis (OA) is a degenerative cartilage disease that is characterized by a local inflammatory reaction. Consequently, many studies have been performed to identify suitable prevention and treatment interventions. In recent years, both arthroscopic microfracture (AM) and stem cell therapy have been used clinically to treat OA. This study aimed to evaluate the clinical effects of AM in the presence and absence of a stromal vascular fraction (SVF) injection in the management of patients with OA. Thirty patients with grade 2 or 3 (Lawrence scale) OA of the knee participated in this study. Placebo group patients (*n* = 15) received AM alone; treatment group patients (*n* = 15) received AM and an adipose tissue‐derived SVF injection. The SVF was suspended in platelet‐rich plasma (PRP) before injection into the joint. Patient groups were monitored and scored with the Western Ontario and McMaster Universities Arthritis Index (WOMAC), Lysholm, Visual Analog Pain Scale (VAS), and modified Outerbridge classifications before treatment and at 6, 12, and 18 months post‐treatment. Bone marrow edema was also assessed at these time points. Patients were evaluated for knee activity (joint motion amplitude) and adverse effects relating to surgery and stem cell injection. Treatment efficacy was significantly different between placebo and treatment groups. All treatment group patients had significantly reduced pain and WOMAC scores, and increased Lysholm and VAS scores compared with the placebo group. These findings suggest that the SVF/PRP injection efficiently improved OA for 18 months after treatment. This study will be continuously monitored for additional 24 months. Stem Cells Translational Medicine *2017;6:187–195*

**Significance Statement**

Arthroscopic microfracture (AM) and stem cell therapy have been used clinically to treat osteoarthritis (OA). This study evaluated the clinical effects of AM in the presence (treatment group) and absence (placebo group) of a stromal vascular fraction (SVF) injection in the knee for OA. The SVF was suspended in platelet‐rich plasma (PRP) before injection. Treatment efficacy differed significantly between placebo and treatment groups. All treatment group patients had significantly improved pain and arthritis index scores compared with the placebo group. These findings suggest that the SVF/PRP injection efficiently improved OA after 18 months. This study will be continuously monitored for 24 months.

## Introduction

Osteoarthritis (OA) is a chronic progressive disease characterized by cartilage degeneration, osteophyte formation, bone reorganization, and loss of joint function 1. OA is the most frequent cause of disability among adults in the United States, and it occurred in >10% of the U.S. adult population in 2009. In 2009, 905,000 knee and hip replacements were carried out in OA patients, costing approximately $42.3 billion in total.

At present, OA is mainly treated with pharmaceuticals 2, 3, hyaluronic acid 4, and neridronate 5, 6. However, these treatments only reduce symptoms and pain or control the inflammation process 7 8 9; none of these drugs actually prevents the progression of OA 10, 11.

Arthroscopic microfracture (AM) has recently gained popularity as a therapy for OA 12 13 14, with some studies reporting significant symptom and functional improvement following the procedure 15. Consequently, AM is indicated as a routine treatment for OA. However, meta‐ and systematic analyses indicate that although AM initially improves OA symptoms 16, 17, this effect is only short term 16. In some cases, particularly among older people, AM can be harmful 16, 18, 19.

As an alternative approach, OA has been treated using platelet‐rich plasma (PRP). PRP contains the pool of cytokines and growth factors stored in platelets 20. Some studies have shown that PRP improves OA symptoms 21, 22. However, this effect has not been not observed for a prolonged period 22 23 24 25 26 27. To improve the effects of PRP, previous studies have investigated the combined injection of PRP with stem cells. Mesenchymal stem cells (MSCs) in conjunction with PRP have been found to mildly improve cartilage healing, and had improved Knee Injury and Osteoarthritis Outcome Score subscores and visual analog pain scores (VAS) compared with PRP‐only therapy 28. Using this approach, it is hypothesized that MSCs differentiate into chondrocytes, which participate directly in cartilage repair and also contribute to immune modulation to inhibit knee joint inflammation.

To date, various stem cell sources have been used to treat OA, such as bone marrow‐derived MSCs (BM‐MSCs) for autograft 29 30 31 32 or allograft 33, adipose‐derived stem cells (ADSCs) 34 35 36, and peripheral blood‐derived stem cells 37 38 39. Other MSC sources include enriched mononuclear cells (MNCs) from bone marrow or umbilical cord blood, stromal vascular fractions (SVFs) from adipose tissue (AT) and purified MSCs obtained from culture‐expanded MNCs.

In their published study, Enea et al. 40 combined autologous bone marrow‐derived cells with microfracture to repair cartilage defects. Their results showed that single‐stage treatment of focal cartilage defects of the knee with microfracture followed by coverage with a polyglycolic acid (PGA)‐hyaluronic acid (HA) matrix augmented with autologous BMCs (PGA‐HA‐CMBMC) was safe and improved knee function. To date, no clinical studies have compared the efficacy of arthroscopic surgery with and without SVF injection in the treatment of OA. This study, therefore, aimed to evaluate the clinical effects of AM alone and in combination with SVF injection on the function and satisfaction of patients with OA.

## Materials and Methods

All experimental protocols were approved by the National Ethical Committee Ministry of Health, Vietnam. This study was registered at clinicaltrials.gov with identifier NCT02142842.

### Inclusion and Exclusion Criteria

All patients enrolled in this study were required to sign a consent form. Patient inclusion criteria were as follows: patients must be older than 18 years, have OA with grade 2 to 3 cartilage degeneration at the time of presentation, failed drug treatment and autologous cartilage transplantation, a Lysholm score less than 65, committed with an artheroplasty condition, and be HIV negative.

A total of 30 patients were enrolled in the study: 15 patients were treated using traditional AM and 15 patients were treated with AM plus an injected mixture of SVF and PRP. The follow‐up time was 18 months for all patients.

### Liposuction

Patients were restricted from taking corticosteroids, aspirin, nonsteroidal anti‐inflammatory drugs and oriental herbal medications for a minimum of 1 week before liposuction. For the liposuction, patients were given spinal anesthesia with 2–3 ml (5 g/L) of bupivacaine hydrochloride. The lower abdomen was also anesthetized. Liposuction was performed using a tumescent solution (500 ml of normal saline and 0.5 ml of 1:1,000 epinephrine). We used a TriPort Harvester cannula (Tulip Medical Products, San Diego, CA, http://www.tulipmedical.com) and a 60‐ml BD Luer‐Lock syringe (BD Biosciences, East Rutherford, NJ, http://www.bd.com) to harvest 100–500 ml of adipose tissue from each patient.

### SVF Isolation

The SVF was isolated from the abdominal adipose tissue of each patient. Approximately 100 ml of lipoaspirate collected from each patient was divided into two 50‐ml sterile syringes. The syringes were stored in a sterile box at 2–8°C and immediately transferred to the laboratory. The SVF was isolated using an ADSC Extraction Kit (GeneWorld, Ho Chi Minh City, Vietnam, http://geneworld.vn) according to the manufacturer's instructions. Briefly, 100 ml of lipoaspirate was placed in a sterile, disposable 250‐ml conical centrifuge tube (Corning Life Sciences, Tewksbury, MA, https://www.corning.com) and washed twice with sterile phosphate‐buffered saline (PBS) by centrifugation at 400*g* for 5 minutes at room temperature. The adipose tissue was then digested using SuperExtract Solution (GeneWorld) containing collagenase at 37°C, for 30 minutes with agitation at 5‐minute intervals. The suspension was centrifuged again at 800*g* for 10 minutes, and the SVF was harvested as a pellet. The pellet was washed twice with PBS to remove any residual enzyme, and resuspended in PBS so that the cell quantity and viability could be measured using an automatic cell counter (NucleoCounter; Chemometec, Lillerød, Denmark, https://chemometec.com).

### Activated PRP Preparation

Activated PRP was derived from the peripheral blood of the same patients as the adipose tissue, using a New‐PRP Pro Kit (GeneWorld) according to the manufacturer's guidelines. Briefly, 20 ml of peripheral blood was collected in vacuum tubes and centrifuged at 800*g* for 10 minutes. The plasma fraction was collected and centrifuged at 1,000*g* for 5 minutes to produce a platelet pellet. Most of the plasma was then removed, leaving 3 ml of plasma for resuspension of the platelets. The inactivated PRP was then activated using activating tubes containing 100 µl of 20% CaCl_2_.

### Preparation of Product for Injection

The final injection product was composed of a mixture of the harvested SVF and activated PRP. Activated PRP was used to dilute the SVF to achieve a suitable dose for injection at 10^7^ SVF cells/ml.

### AM and SVF/PRP Injection

All patients in both groups received AM, which was used to confirm the degree of OA in each patient. Local chondral lesions were removed using medical instruments and an arthroscopic shaver. Microfractures were performed in accordance with the methods described by Steadman et al. 41. The 30 patients were grouped into a treatment group and a placebo group (*n* = 15 per group). After arthroscopic marrow stimulation by AM, the water flow was stopped and excess water was aspirated from the joint cavity. In the treatment group, the SVF and activated PRP mixture (5 ml per knee) was injected. Patients in the placebo group were injected with saline.

### Follow‐Up and Evaluation

Patients were monitored in the hospital for 1 week postinjection. During this time, all complications, including shock, infection, and inflammation, were noted. After this, patients were followed for 18 months. Western Ontario and McMaster Universities Arthritis Index (WOMAC), Lysholm, and VAS scores were assessed 1, 6, 12, and 18 months after surgery. Radiographic imaging and magnetic resonance imaging (MRI) were performed 6 and 12 months post‐treatment. In this study, we used the modified VAS scores. with 4 indicating no pain; 3, mild pain; 2, moderate pain; 1, severe pain; and 0, worst pain possible.

Patients began continuous passive motion 4–5 days post‐treatment. Partial weight bearing was permitted at 2 weeks, progressing to full weight bearing 4 weeks after surgery. Isometric quadriceps and hamstring training with straight‐leg raises were advised during the non‐weight‐bearing period. Light sport activities such as swimming, cycling, or jogging on even, soft ground were permitted at 6 months. Permission to participate in unrestricted sports activity was given after 12 months.

### Statistical Analysis

Results were expressed as the mean ± SD. One‐way analysis of variance and two‐tailed *t* tests were used for all statistical analyses, which were performed with GraphPad Prism 4.0 (GraphPad Software, La Jolla, CA, https://www.graphpad.com). *p* values <.05 were considered statistically significant.

## Results

### Patient Characteristics

This study was performed from April 2013 to September 2015 at two hospitals (Van Hanh General Hospital and 115 Hospital, both in Ho Chi Minh City, Vietnam). The 30 patients who satisfied the study standard were divided into 2 groups: placebo (*n* = 15) and treatment (*n* = 15). Demographic analysis found that these groups had an equivocal age, body mass index, sex, and Kellgren‐Lawrence OA grade (Table 1). The Kellgren‐Lawrence grade was based on x‐rays, and was confirmed during AM ( supplemental online Fig. 1).

**Table 1:** Study participant demographic characteristics

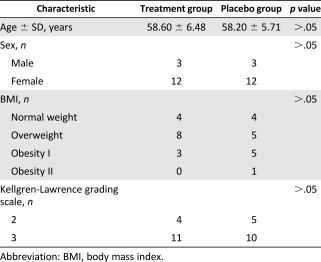

### Adverse Effects

No adverse events were observed during the study in either group. We identified four cases with complications not related to the AM or SVF injection; these complications included high blood pressure, chest pain, dyspnea, and urinary retention.

### Changes in WOMAC Scores

**Figure 1:**
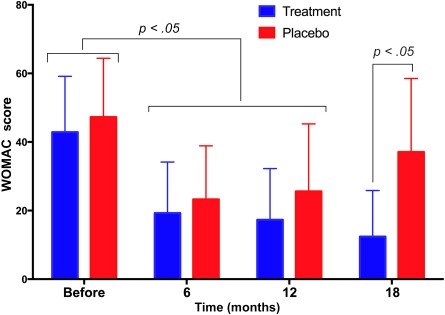
WOMAC scores in placebo and treatment groups at 6, 12, and 18 months post‐treatment. After 6 months, WOMAC scores significantly decreased in both the treated and placebo groups. At 12 and 18 months, WOMAC scores continued to decrease in the treatment group and increased in the placebo group. Abbreviation: WOMAC, Western Ontario and McMaster Universities Arthritis Index.

Figure 1 shows the WOMAC score results. Pretreatment WOMAC scores were equivocal, with a small nonsignificant difference observed between the placebo and treatment group (47.27 ± 17.13 vs. 42.87 ± 16.29, respectively; *p* > .05). At 6 and 12 months after treatment, the WOMAC scores in both groups significantly decreased compared with the pretreatment scores. In the placebo group, WOMAC scores decreased from 47.27 ± 17.13 to 23.27 ± 15.61 and 25.60 ± 19.69 at 6 and 12 months after surgery, respectively. In the treatment group, WOMAC scores decreased from 42.87 ± 16.19 to 19.27 ± 14.87 and 17.33 ± 14.91 at 6 and 12 months after surgery, respectively. At 6 and 12 months after surgery, the differences in the WOMAC scores between the treatment and placebo groups were nonsignificant (*p* > .05). However, a slight difference was observed between the 2 groups 12 months after surgery. WOMAC scores in the treatment group gradually decreased at 6 and 12 months compared with the pretreatment scores, although the WOMAC score 12 months after surgery was slightly increased compared with the score 6 months after the procedure.

The difference in the WOMAC scores of the placebo and treatment groups became more pronounced after 18 months of monitoring. In the placebo group, the WOMAC score increased from 25.60 ± 19.69 at 12 months to 37.08 ± 21.45 at 18 months. More importantly, WOMAC scores at 18 months in the placebo group were not significantly different compared with pretreatment scores. The WOMAC scores of the treatment group decreased at 6, 12, and 18 months (19.27 ± 14.87, 17.33 ± 14.91, and 12.40 ± 13.44, respectively) after surgery compared with the pretreatment score (42.87 ± 16.29). The 18‐month WOMAC scores were also significantly different between the placebo and treatment groups (*p* < .05; Fig. 1).

### Changes in Lysholm Scores

**Figure 2:**
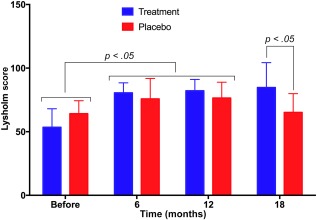
Lysholm scores in placebo and treatment groups at 6, 12, and 18 months post‐treatment. In both treated and placebo groups, the Lysholm score significantly increased at 6 months post‐treatment. At 12 and 18 months post‐treatment, the Lysholm scores of the treatment group continued to increase, whereas those of the placebo group gradually decreased.

The results presented in Figure 2 show that Lysholm scores changed in both the treatment and placebo groups, but in opposite directions. The Lysholm scores increased significantly in both groups 6 months post‐treatment compared with the pretreatment score (*p* < .05). In the placebo group, however, the Lysholm scores were decreased dramatically 18 months after surgery to a level comparable to the pretreatment score (75.80 ± 16.05, 76.47 ± 12.44, and 65.17 ± 14.74 at 6, 12, and 18 months, respectively, compared with 64.13 ± 10.19 pretreatment). In the treatment group, the Lysholm scores gradually increased over 6, 12, and 18 months compared with pretreatment scores (80.53 ± 7.86, 82.13 ± 8.98, 84.73 ± 19.54, and 53.47 ± 14.56, respectively). At 18 months, the mean Lysholm score of the placebo and treatment groups was significantly different (*p* < .05).

### Changes in VAS Scores

**Figure 3:**
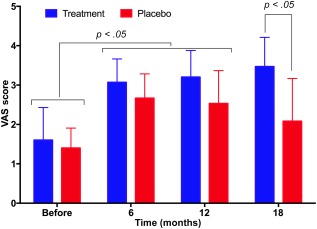
VAS scores at pretreatment and 6, 12, and 18 months post‐treatment in the placebo and treatment groups. VAS scores in the treatment group gradually increased post‐treatment. In the placebo group, scores increased after 6 months and gradually decreased at 12 and 18 months. Abbreviation: VAS, Visual Analog Pain Scale.

Similar to the Lysholm scores, VAS scores in both the treatment and placebo groups changed, but in opposite directions (Fig. 3). In the placebo group, VAS scores significantly increased after 6 months compared with those at pretreatment (2.67 ± 0.62 vs. 1.40 ± 0.51, respectively; *p* < .05). However, the scores then decreased from 12 to 18 months (2.53 ± 0.83 and 2.08 ± 1.08, respectively). In the treatment group, VAS scores continuously increased from 1.60 ± 0.83 at pretreatment to 3.01 ± 0.59, 3.20 ± 0.68, and 3.47 ± 0.74 at 6, 12, and 18 months, respectively (*p* < .05).

### Cartilage Injury Evaluation by MRI

**Figure 4:**
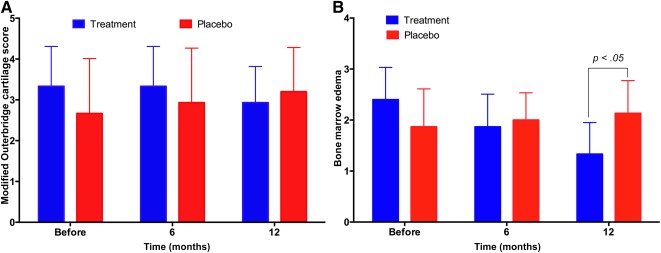
OS and BME scores at pretreatment and 6 and 12 months post‐treatment. Although the changes were nonsignificant, OS scores increased in the placebo group and decreased in the treatment group **(A);** and BME was significantly decreased in the treatment group 12 months after surgery, and only slightly increased in the placebo group **(B)**. Abbreviations: BME, bone marrow edema; OS, Outerbridge classification system.

Based on the MRI results and the Outerbridge classification system (OS), changes in cartilage injury were recorded and are presented in Figure 4A. OS scores gradually increased in the placebo group from pretreatment to 6, 12, and 18 months post‐treatment (2.67 ± 1.35, 2.93 ± 1.34, and 3.20 ± 1.08, respectively). However, scores decreased in the treatment group from pretreatment to 12 months post‐treatment (3.33 ± 0.97 vs. 2.93 ± 0.88, respectively).

Although differences in OS scores were nonsignificant (*p* > .05), the trend was clearly different between the two groups: OS scores increased in the placebo group over time but decreased in the treatment group. MRI imaging demonstrated that the cartilage layer was thicker in the treatment group 12 months after AM ( supplemental online Fig. 3).

### Bone Marrow Edema

Bone marrow edema (BME) was also recorded based on the MRI results. The results presented in Figure 4B and supplemental online Figure 2 show that BME was considerably deceased 12 months after surgery in the treatment group, although it was moderately increased in the placebo group. In the treatment group, BME gradually decreased from pretreatment to 6 and 12 months post‐treatment (2.40 ± 0.63, 1.86 ± 0.64, and 1.33 ± 0.62, respectively), with a significant difference at 12 months (*p* < .05).

In the placebo group, BME increased moderately at 6 to 12 months post‐treatment compared with pretreatment measurements (1.87 ± 0.74 at pretreatment vs. 2.00 ± 0.53; 2.13 ± 0.64 at 6 to 12 months post‐treatment, respectively).

### Correlating OA Stage With Treatment Efficacy

Although the number of patients included in this study was low, we were able to evaluate the relative efficacy of AM plus SVP/PRP treatment between patients with stage 2 (*n* = 4) and stage 3 (*n* = 11) OA.

**Figure 5:**
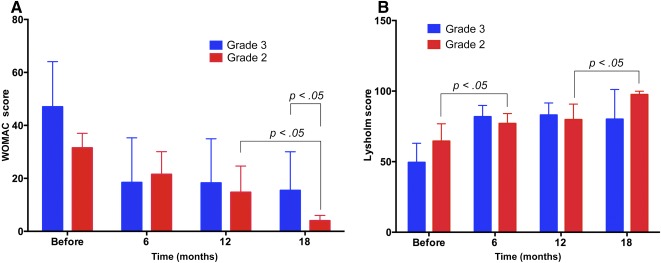
WOMAC and Lysholm scores in stage 2 and 3 osteoarthritis (OA; treatment group). Stromal vascular fraction and platelet‐rich plasma injection significantly improved WOMAC and Lysholm scores in patients with stage 2 OA compared with those with stage 3 disease. Abbreviations: OA, osteoarthritis; WOMAC, Western Ontario and McMaster Universities Arthritis Index.

The results presented in Figure 5A and 5B shows that the SVF/PRP injection affected patients with stage 2 and 3 OA differently with respect to both WOMAC and Lysholm scores, with significant differences observed at 18 months post‐treatment. Although the WOMAC and Lysholm scores were significantly improved in both stage 2 and 3 OA groups at 18 months post‐treatment compared with pretreatment, only in stage 2 OA patients were both WOMAC and Lysholm scores significantly improved at 18 months compared with 12 months post‐treatment (*p* < .05).

**Figure 6:**
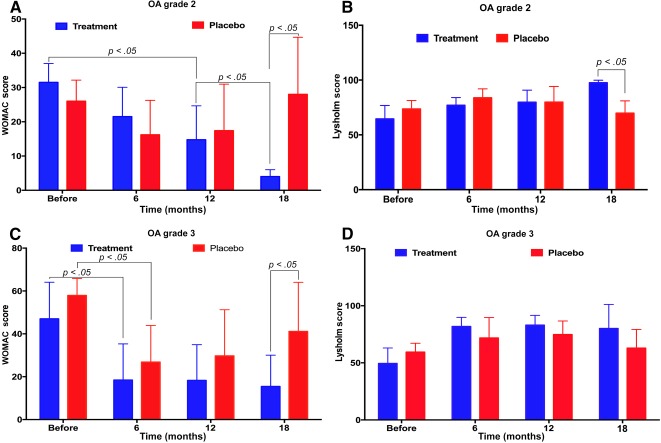
WOMAC and Lysholm scores in stage 2 and 3 OA treated and placebo groups. Stage 2 patients improved more rapidly compared with stage 3 patients. Abbreviations: OA, osteoarthritis; WOMAC, Western Ontario and McMaster Universities Arthritis Index.

When we separately compared the stage 2 and stage 3 treatment groups with the placebo group, the differences became clearer (Fig. 6). Compared with the stage 2 OA members of the placebo group, the stage 2 treatment group had significantly improved WOMAC and Lysholm scores. Compared with the stage 3 OA placebo group, the stage 3 treatment group was improved but to a lesser extent. Patients in the stage 2 treatment group continuously improved in both their WOMAC and Lysholm scores at 12 and 18 months post‐treatment, whereas the improvement rate was slower in the stage 3 OA group.

### Changes in Knee Joint Function

The knee joint function of treated patients was significantly improved at 18 months post‐treatment, and their joint motion amplitude (JMA) increased from 116.2 ± 27.1 at pretreatment to 138.8 ± 12.0 at 18 months post‐treatment. JMA also increased in the placebo group from 120.6 ± 24.3 pretreatment to 133.3 ± 17.9 at 18 months post‐treatment but to a lesser extent than in the treatment group.

## Discussion

AM is the conventional method to treat cartilage degeneration, including OA lesions. However, the benefits of AM are gradually lost in the 18 months following treatment. This study aimed to combine the AM approach with an injection of SVF and PRP to improve treatment efficacy. Autologous ADSCs and autologous PRP from the peripheral blood were used in this study. Although previous studies used allogeneic‐derived MSCs to effectively improve OA, we used an autologous source to minimize the side effects relating to host factors, specifically inflammation.

Both SVF and ADSCs (the purified form of SVF) have been used clinically in the treatment of conditions such as multiple sclerosis 42, femoral head necrosis 43, 44, chronic myocardial ischemia 45, critical limb ischemia, progressive supranuclear palsy 46, and acute respiratory distress syndrome 47. Our results indicate that AM with a combined SVF/PRP injection significantly improved and prolonged the treatment efficacy of AM for OA. At 6 months post‐treatment, the WOMAC, Lysholm, and VAS scores were significantly improved compared with pretreatment scores. These scores were further and significantly improved at 12 and 18 months post‐treatment in the SVF/PRP group. Some of the patients obtained scores similar to that of healthy individuals. The WOMAC is a widely used, proprietary set of standardized questionnaires used by health professionals to evaluate the condition of patients with OA of the knee and hip, including pain, stiffness, and joint function. Higher WOMAC scores correspond with a higher level of pain, stiffness, and functional limitation. In the treatment group, the mean WOMAC score was 12.40 ± 13.44 at 18 months after surgery. The WOMAC Index is sensitive to change and, therefore, is considered a suitable scale to assess OA.

In addition to the WOMAC Index, the Lysholm scale is one of the most commonly used scoring systems for measuring OA. It was first published in 1982 and comprises 8 questions designed to evaluate joint instability in younger patients. This scale measures disability and focuses on the patient's perception of their ability to perform activities of daily living, as well as various intensities of physical activity 48. According to this scale, a score of 84–90 is considered a good result. The average Lysholm score of patients in the AM plus SVF/PRP group 18 months after treatment was 84.73 ± 19.54.

Supporting the change seen in the WOMAC and Lysholm scores, the VAS scale scores also showed clear improvements in the treatment group. The VAS is a psychometric response scale that can be used in questionnaires. It is a measurement approach for subjective characteristics or attitudes that cannot be directly measured. The VAS scale for pain is divided into 4 points: 4 (no pain), 3 (mild pain), 2 (moderate pain), and 0 (severe pain). The WOMAC, Lysholm, and VAS scores demonstrated that at 18 months post‐treatment, all patients in the treatment group had significantly improved pain, movement, and capacity for physical activity. Some patients’ scores appeared similar to those of healthy individuals.

AM resulted in significantly reduced pain and improved knee function 6 months after the procedure, and these persisted for up to 12 months. However, by 18 months post‐AM, the symptoms of OA in the majority of patients reverted back to pretreatment levels. These results support those of several published studies. Thorlund et al. 16 reviewed 1,789 reports of AM used in degenerative knees. They found that AM had a small, inconsequential benefit in the management of OA, was effective for a limited time, and any benefits were absent 1 to 2 years after surgery. Furthermore, in patients with moderate to severe OA of the knee, Risberg 18 showed that the addition of arthroscopy to a regimen of physiotherapy and medication did not improve the physical function, pain, or health‐related quality of life of patients with OA.

Our results showed that SVF in combination with PRP significantly improved the outcomes of AM for OA of the knee. SVF and PRP not only maintained and prolonged the effects of AM, but also increased overall treatment efficacy. All WOMAC, Lysholm, and VAS scores were noticeably improved compared with AM alone at 6 and 12 months post‐treatment.

From the MRI results, we showed that OS scores and BME were significantly improved at 12 months post‐treatment. Whereas OS scores and BME improved after AM in the placebo group, both of these indicators were decreased in the treatment group. In particular, BME was significantly decreased at 12 months post‐treatment. OS classification is a grading system for joint cartilage breakdown: grade 0 represents normal joint cartilage; grade 1 represents cartilage with softening and swelling; grade 2 represents a partial‐thickness defect with fissures on the surface that do not reach the subchondral bone or exceed 1.5 cm in diameter; grade 3 represents fissuring to the level of subchondral bone in an area with a diameter more than 1.5 cm; and grade 4 represents exposed subchondral bone. Our results showed that the OS scores decreased from 3.33 ± 0.97 pretreatment to 2.93 ± 0.88 at 12 months post‐treatment in the treatment group. These results showed that the cartilage layer was thicker 12 months after the knee was injected with SVF and PRP, a finding congruent with our previously published study 36. Other studies have shown that SVF in combination with PRP stimulates cartilage regeneration, with a thicker cartilage layer observed using post‐treatment MRI evaluation 34, 44. We have shown in a mouse model that SVF and PRP can stimulate knee cartilage regeneration 49. The impact of a SVF/PRP injection in our study was also similar to effects noted in canine 50 51 52, rabbit 53, 54, horse 55, rat 56, and goat 57 models. Cartilage regeneration in these models was attributed to neocartilage triggered by SVF and PRP. In a rabbit model, Dragoo et al. 58 showed that autologous ADSCs were able to re‐establish the joint surface in rabbits. They found neocartilage was present in 100% of treated rabbits (12 of 12), whereas only 8% of control rabbits (1 of 12) had neocartilage.

The mechanisms of action of SVF and ADSCs have been investigated in previous studies. In 2003, Gimble and Guilak 57 showed that injected ADSCs were able to protect and heal injured cartilage. Other benefits of ADSCs have been reported for cartilage regeneration, including anti‐inflammatory properties 59, 60 and immune modulation. ADSCs can produce and secrete cytokines and growth factors that can trigger chondrogenesis, including transforming growth factor‐β (TGF‐β), bone morphogenic protein 2 (BMP‐2), BMP‐4, BMP‐7, insulin‐like growth factor 1, and fibroblast growth factor 2 (FGF‐2). ADSCs also produce cytokines that modulate the recipient immune system, including TGF‐β, hepatocyte growth factor, nitric oxide, indolamine‐2,3‐dioxygenase, TNF‐α 61 and interferon‐γ 62, 63. In vitro, cultured ADSCs suppress the host's immune response and the T‐cell proliferation as effectively as do BM‐MSCs 61, 64. Further studies have demonstrated that ADSCs actually stimulate a lesser proliferative response than do allogeneic PBMCs, but a similar response to BM‐MSCs 65 66 67. These findings suggest that ADSCs can replace BM‐MSCs in the field of regenerative medicine 61.

The anti‐inflammatory roles of ADSCs and PRP were also confirmed in our study by the obvious improvement of BME in the treatment group. BME is a condition characterized by the accumulation of excessive fluid in bone marrow‐related structures. BME is a predictor for the progression of knee OA in the compartment ipsilateral to the bone marrow lesion 68. BME was significantly reduced and the cartilage layer thickness was increased in the SVF/PRP‐treatment group, indicating that OA was significantly improved. The increased BME observed in the placebo group may have been related to the progression of OA and inflammation after AM.

Cartilage regeneration in OA knees following AM and the combined SVF/PRP injection was likely because of the combination of SVF and PRP. However, SVF is likely to be the main contributor to this healing response. PRP has been used to treat knee OA in previous studies 69 70 71, but almost all of these studies showed that PRP significantly reduced short‐term pain without concurrent cartilage regeneration 21, 69, 71, 72. In combination with ADSCs, PRP can improve chondrogenesis in vitro and in vivo 73. The components of PRP play important roles in stimulating grafted and endogenous cell growth and differentiation. PRP contains at least six known growth factors, including: platelet‐derived growth factor, which promotes blood vessel growth and cell division; TGF‐β, which promotes cell mitosis and bone metabolism; vascular endothelial growth factor, which promotes blood vessel formation; epidermal growth factor, which promotes cell growth and differentiation, angiogenesis, and collagen formation; FGF‐2, which promotes cell differentiation and angiogenesis; and IGF, which is a regulator of all of the body's cell types 74 75 76.

We also observed that the regeneration response of cartilage to injected SVF/PRP was different between patients with grade 2 and 3 OA. Both WOMAC and Lysholm scores showed that the recovery of patients with grade 2 OA was faster than that of those with grade 3 disease. In particular, the improvement of WOMAC and Lysholm scores in patients with OA grade 2 were significant at 18 months compared with 12 months post‐treatment. This demonstrated that OA grade 2 was treated with higher efficacy than OA grade 3 following SVF/PRP injection. Although this study was limited with respect to the sample size of patients with either grade 2 or 3 OA, these results are similar to other treatment options for OA, such as HA and PRP injections 24, 25.

Finally, JMA was compared between treated and placebo group patients. JMA was clearly increased in the treatment group compared with the placebo group, which agrees with both our subjective and radiographic analyses. More importantly, almost all patients in the treatment group exhibited a JMA similar to healthy individuals. The mean JMA was 138.8 ± 12 at 18 months post‐treatment. The mean JMA of healthy individuals has been reported to be 140.0 (range, 113.9–166.4) 77.

We believe that our study is the first to evaluate AM with and without SVF for OA treatment with an 18‐month follow‐up time. Although Freitag et al. 78 recently performed a similar study to ours, their follow‐up time was only 12 months.

## Conclusion

This study showed that AM with SVF/PRP injection was effective for knee OA and had better long‐term outcomes than AM alone. Our preliminary analysis also showed that grade 2 knee OA was improved to a greater extent than grade 3 disease following AM with SVF injection. AM with SVF injection significantly improved WOMAC, Lysholm, and VAS scores over the entire 18‐month study period. MRI findings showed that the regenerated cartilage layer of patients treated with AM and SVF was thicker at 12 and 18 months after the procedure. Furthermore, the JMA of SVF/PRP‐treatment patients 18 months after surgery was significantly improved and comparable with that of healthy individuals. No adverse effects were recorded in any treated patients. From these findings, we conclude that AM with SVF/PRP injection may be a suitable treatment for grade 2 and 3 OA of the knee.

## Author Contributions

P.D.N.: conception and design, administrative support, provision of study material or patients; T.D.‐X.T., H.T.‐N.N., and H.T.V.: provision of study material or patients, collection and/or assembly of data; P.T.‐B.L.: conception and design, administrative support, provision of study material or patients, collection and/or assembly of data; N.L.‐C.P. and N.B.V.: provision of study material or patients; N.K.P.: conception and design, data analysis and interpretation; P.V.P.: conception and design, data analysis and interpretation, manuscript writing, final approval of manuscript.

## Disclosure of Potential Conflicts of Interest

The authors indicated no potential conflicts of interest.

## Supporting information

Supporting Information
