# Supplementary material for: Comparative Clinical Observation of Arthroscopic Microfracture in the Presence and Absence of a Stromal Vascular Fraction Injection for Osteoarthritis
Source: Stem Cells Transl Med. 2016 Aug 29;6(1):187–95. doi: 10.5966/sctm.2016-0023 (PMC5442736; doi:10.5966/sctm.2016-0023)
Supplement: Supplementary file 1 — Supporting Information [file SCT3-6-187-s001.pdf]

Supplemental Figures – Pham et al.

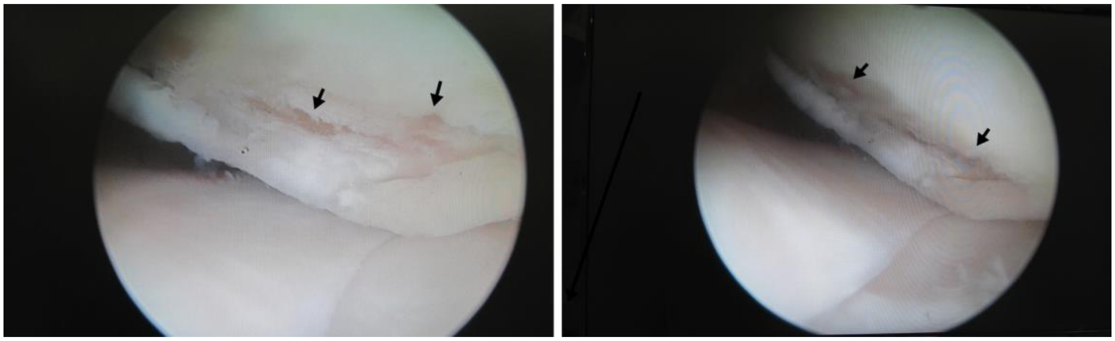

Figure S1: Arthroscopic microfracture of a patient with Grade III OA (from patient N.T.C 63 yrs old). Arrows indicate injured cartilage sites.

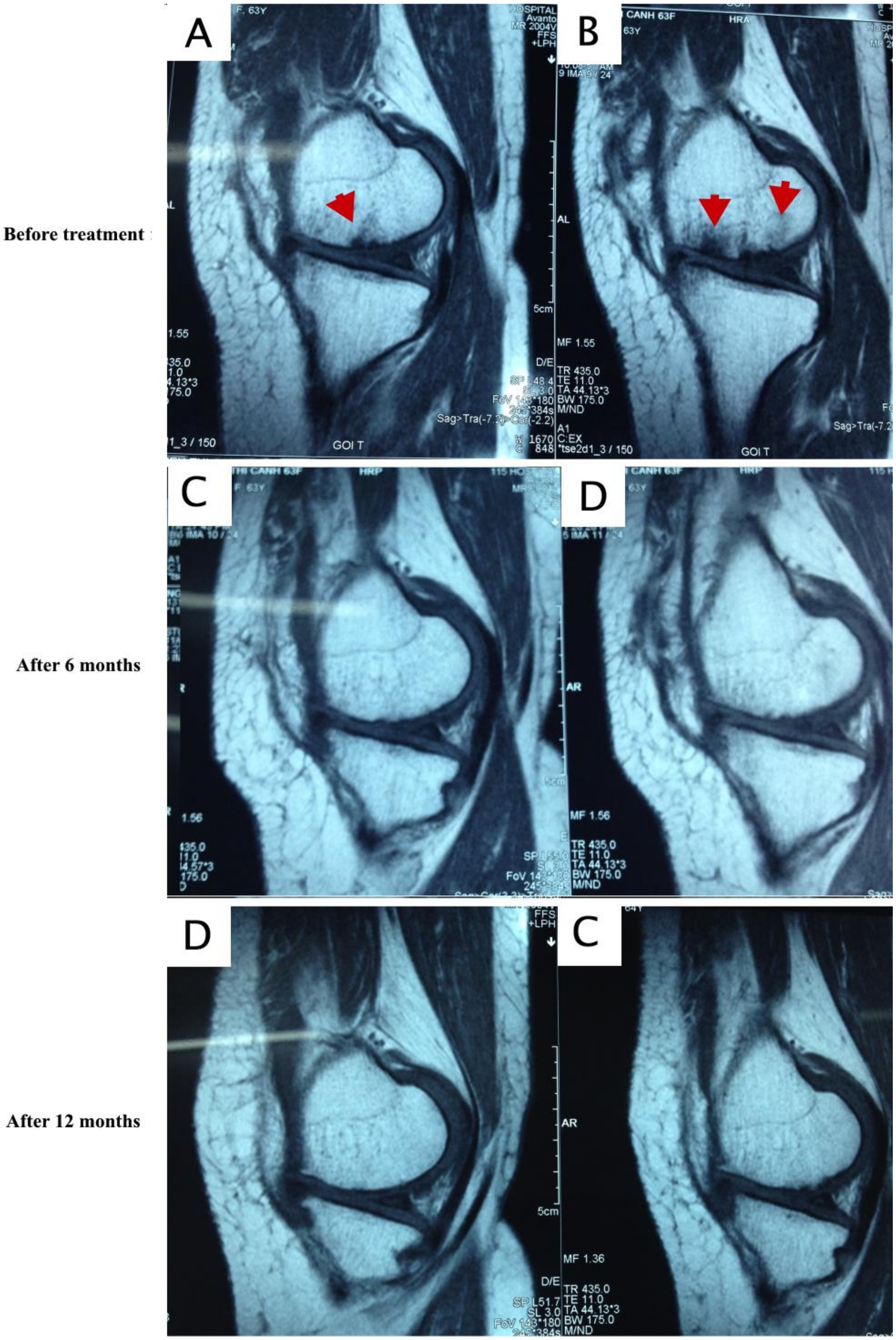

Figure S2: Bone marrow edema was significantly improved 6 and 12 months after treatment. MRI results of a patient (N.T.C 63 yrs old) in the treatment (AM and SVF injection) group pre-treatment (A,B) and at 6 (C,D) and 12 (E,F) months post-treatment. The arrows showed that patients with bone marrow edema before treatment (A, B), and bone marrow edema gradually decreased after treatment (C, D, E, F).

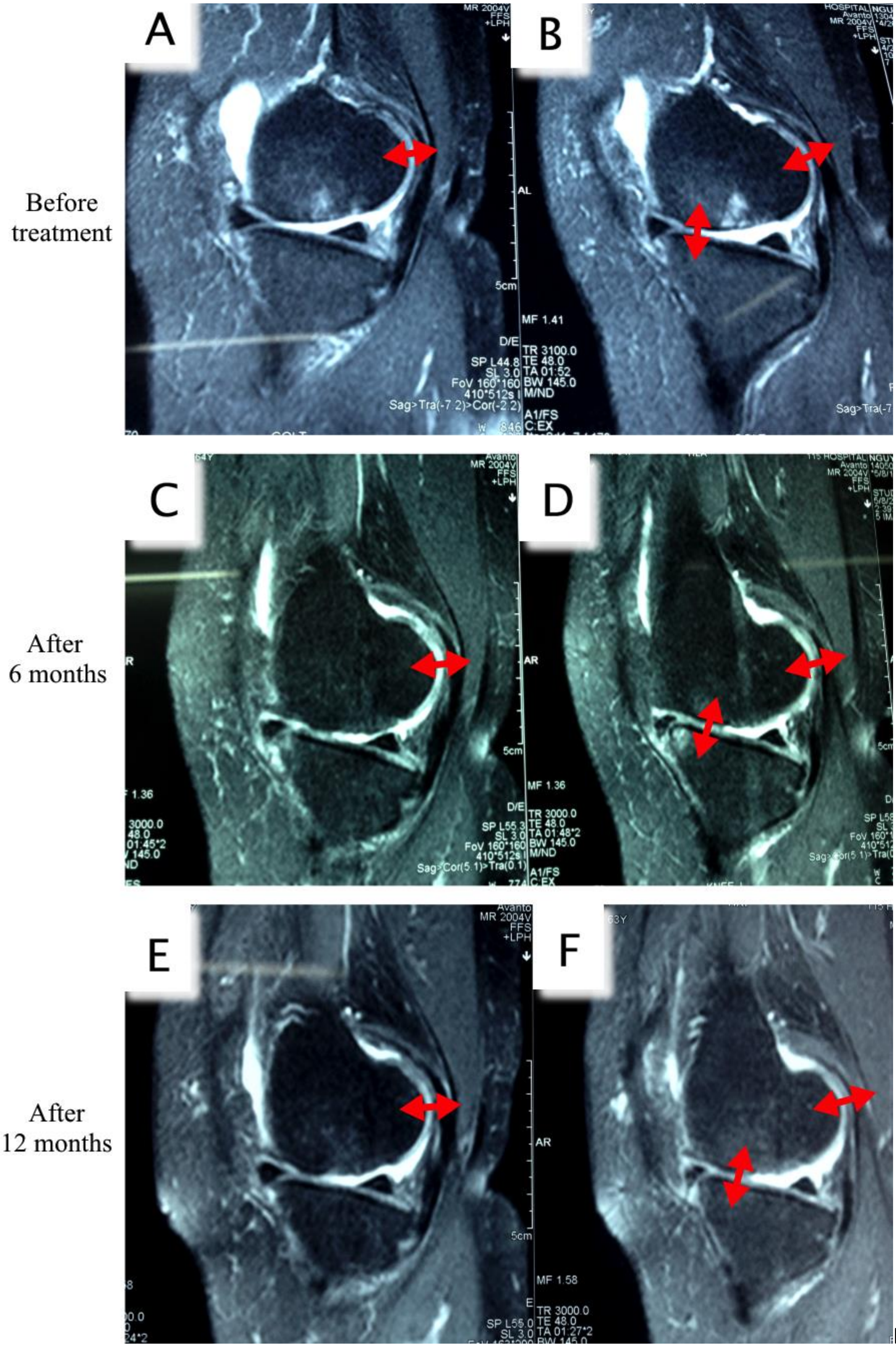

Figure S3: The cartilage layer of the treatment group was thicker 6 and 12 months after surgery. MRI results of a patient (N.T.C 63 yrs old) in the treatment group pre-treatment (A, B) and at 6 (C,D) and 12 (E,F) months post-treatment. The MRI results showed that the cartilage layers were thicker after 6 (C,D) and 12 (E,F) months of treatment with AM and SVF injection.
